# Fibroblast‐growth‐factor‐23 in heart failure with preserved ejection fraction: relation to exercise capacity and outcomes

**DOI:** 10.1002/ehf2.13020

**Published:** 2020-09-16

**Authors:** Prathap Kanagala, Jayanth R. Arnold, Jamal N. Khan, Anvesha Singh, Gaurav S. Gulsin, Mohamed Eltayeb, Pankaj Gupta, Iain B. Squire, Gerry P. McCann, Leong L. Ng

**Affiliations:** ^1^ Department of Cardiovascular Sciences University of Leicester, National Institute for Health Research (NIHR) Leicester Biomedical Research Centre Leicester UK; ^2^ Aintree University Hospital Liverpool UK; ^3^ Liverpool Centre for Cardiovascular Science Liverpool UK

**Keywords:** FGF23, Bone, Exercise capacity, Heart failure with preserved ejection fraction, Inflammation, Prognosis

## Abstract

**Aims:**

This study aimed to assess plasma fibroblast growth factor 23 (FGF23) in patients with heart failure with preserved ejection fraction (HFpEF) and its relation to inflammation, renal function, clinical and imaging characteristics, exercise capacity, and prognosis.

**Methods and results:**

We performed a prospective, observational study of 172 age‐matched and sex‐matched subjects (HFpEF *n* = 130; controls *n* = 42, age 73 ± 9, female 50%) who underwent plasma biomarker sampling, echocardiography, cardiac magnetic resonance imaging, and 6 min walk testing (6MWT). The primary endpoint was the composite of all‐cause death or HF hospitalization. FGF23 was higher in HFpEF compared with controls (62 [42–105] vs. 34 [22–41] pg/mL, *P* < 0.0001). In HFpEF, FGF23 correlated with greater symptom burden (New York Heart Association class: *r* = 0.308), poorer exercise capacity (6MWT distance: *r* = −0.345), and plasma biomarkers reflecting inflammation (highly sensitive C‐reactive protein: *r* = 0.207, myeloperoxidase: *r* = 0.311), bone metabolism (osteoprotegerin: *r* = 0.446), renal dysfunction (urea: *r* = 0.267, creatinine: *r* = 0.351, estimated glomerular filtration rate: *r* = −0.367), and echocardiographic E/e′ (*r* = 0.298); *P* < 0.05. Following multivariable linear regression modelling, FGF23 remained independently associated with shorter 6MWT distance (*P* = 0.012) in addition to age, body mass index, and lower haemoglobin. During follow‐up (median 1428 days), there were 61 composite events (21 deaths, 40 HF hospitalizations) in patients with HFpEF. In multivariable Cox regression analysis, FGF23 [adjusted hazard ratio (HR) 1.665; 95% confidence interval (CI) (1.284–2.160; *P* < 0.0001)], B‐type natriuretic peptide (HR 1.433; CI 1.053–1.951; *P* = 0.022), and prior HF hospitalization (HR 2.058; CI 1.074–3.942; *P* = 0.030) were independent predictors of the composite endpoint.

**Conclusions:**

Plasma FGF23 is higher in HFpEF compared with age‐matched and sex‐matched controls and is strongly associated with exercise incapacity and prognosis. FGF23 correlates with plasma markers of inflammation and renal impairment.

## Introduction

Heart failure (HF) is an increasingly prevalent and important health care issue. Of incident cases of HF, approximately half are HF with preserved ejection fraction (EF) (HFpEF), a proportion which has increased over recent years and which is likely to be the predominant phenotype in the near future.^1^ While the cause of HF with reduced EF (HFrEF) is usually evident, and management is based upon a large evidence base,^2^ the aetiology of HFpEF is often unclear, and there are no specific evidence‐based treatments.^3^

HFpEF includes a heterogeneous patient population, which have in common a typical phenotype, encompassing preserved left ventricular (LV) function and abnormal cardiac structure in the form of LV hypertrophy (LVH) and atrial dilatation, with elevated circulating natriuretic peptides. It is increasingly recognized that a multitude of co‐morbidities, singly or more often in combination, may contribute to the development of the HFpEF syndrome^3^; these include not only structural myocardial factors but also abnormalities of microvascular and endothelial function possibly driven by inflammation and myocardial energetics.^4^ Whatever the factors contributing to the syndrome of HFpEF in the individual patient, the condition is invariably characterized by exercise intolerance, the pathophysiological mechanisms of which are unclear. Fibroblast growth factor (FGF) 23 is a regulator of bone mineral metabolism circulating in elevated levels in patients with chronic kidney disease, in whom it is associated with mortality risk and with progressive renal impairment.^5^

Circulating FGF23 levels have been associated with risk of incident cases of HFpEF in community‐based populations^6^ and of mortality risk in HFrEF.^7^ Little is known about the role of FGF23 in HFpEF. In mouse models, FGF23 induces LVH via activation of the FGF receptor 4 (FGFR4)^8^, ^9^; the observation that blockage of FGFR4 attenuates this response^10^ suggests a possible direct role for FGF23 in the development of LVH and thus a possible contribution to the HFpEF phenotype.

The aims of our study were to identify whether FGF23 was associated with plasma markers of inflammation, renal dysfunction, clinical metrics of disease severity including exercise capacity, imaging characteristics, and prognosis in patients with HFpEF undergoing extensive tissue characterization with cardiac magnetic resonance (CMR).

## Methods

### Study population

As reported previously,^11^, ^12^, ^13^ subjects with a clinical diagnosis of HFpEF were recruited as part of a prospective, observational, cohort study conducted in a single, tertiary‐cardiac centre. The inclusion criteria for HFpEF were as follows: clinical or radiographic evidence of HF, LVEF > 50% on transthoracic echocardiography (TTE) and age ≥ 18 years. Exclusion criteria were as follows: documented myocardial infarction (MI) in the preceding 6 months, suspected or confirmed cardiomyopathy [e.g. hypertrophic cardiomyopathy (HCM) amyloid] or constrictive pericarditis, severe native valve disease, non‐cardiovascular life expectancy < 6 months, severe pulmonary disease (forced expiratory volume < 30% predicted or forced vital capacity < 50% predicted), estimated glomerular filtration rate (eGFR) < 30 mL/min/m^2^, and standard contraindications to CMR imaging.

HFpEF patients were compared with asymptomatic age‐matched and sex‐matched controls without known cardiac disease. The control population did include individuals with a history of hypertension (50%), because this is highly prevalent in the general population as well as in HFpEF, and the aim for our study was to identify factors differentiating between patients with and without HFpEF.

During a single study visit, all subjects underwent clinical assessment of New York Heart Association (NYHA) status, blood sampling, TTE, CMR, standardized 6 min walking test (6MWT),^14^ and Minnesota Living with Heart Failure (MLHF) questionnaire^15^ evaluation. The study conformed with the principles outlined in the *Declaration of Helsinki*. The research protocol was approved by the UK National Research Ethics Service (reference: 12/EM/0222). Written informed consent was obtained from all subjects prior to participation. The study was registered on Clinicaltrials.gov (NCT03050593).

### Plasma sampling and analysis

At recruitment, blood sampling was undertaken for B‐type natriuretic peptide (BNP; immunoassay, Siemens, Erlangen, Germany), haematocrit, haemoglobin, and renal function and assayed in our hospital laboratory.

Blinded single batch testing of FGF23, osteoprotegerin (OPG), highly sensitive C‐reactive protein (hs‐CRP), and myeloperoxidase (MPO) was undertaken from residual supernatant plasma stored at −80°C in cryotubes using a Luminex® bead‐based multiplex assay,^16^ enabling high‐throughput biomarker profiling as previously described.^17^

### Transthoracic echocardiography

Echocardiography (iE 33 System, Philips Medical Systems, Best, the Netherlands) was performed as previously detailed.^11^, ^12^, ^13^ For study inclusion, LVEF was calculated using the biplane method or visually estimated in subjects with poor endocardial border definition. Trans‐mitral Doppler was used to measure the early diastolic inflow E wave. Tissue Doppler measured medial and lateral mitral annular tissue velocities (e′) were averaged to derive E/e′ as an overall measure of diastolic dysfunction.

### Cardiac magnetic resonance

The CMR protocol used has previously been detailed.^11^, ^12^, ^13^ All scans were performed on a 3 Tesla platform (Siemens Skyra, Erlangen, Germany). In brief, the protocol comprised the following: standard breath‐held steady‐state free precession long‐axis and short‐axis cine imaging; short‐axis pre‐contrast and post‐contrast T1 maps; and late gadolinium enhancement (LGE) imaging. The total contrast dose administered was 0.15 mmol/kg of Gadovist (Bayer Healthcare, Berlin, Germany).

All CMR analyses were performed by a single observer (P. K.) blinded to clinical data, using *CVI42* software (Circle Cardiovascular Imaging, Calgary, Canada). LV volumes, EF, and mass (excluding papillary muscles) were calculated from the short‐axis cine stack.^11^, ^12^ Left atrial (LA) volumes and EF (LAEF) were derived from the biplane method, excluding the appendage and pulmonary veins.^11^ All volumetric and mass data were indexed to body surface area (BSA). LGE was assessed qualitatively for the presence and pattern of focal fibrosis and categorized as MI or non‐MI fibrosis, requiring consensus by two experienced observers (P. K. and G. P. M.). Extracellular volume (ECV) and ECV indexed to BSA (iECV) were also calculated as measures of diffuse myocardial fibrosis from mid‐ventricular T1 maps, as reported recently by our group with excellent reproducibility.^12^

### Follow‐up and endpoints

The primary endpoint was a composite of all‐cause mortality or hospitalization for HF (defined as a hospital admission for which HF was the primary reason and necessitating diuretic, inotropic, or intravenous nitrate therapy). Only first events were included in the outcome analysis. All subjects underwent a minimum of 1 year follow‐up, post‐study entry. Outcome data were sourced from hospital records.

### Statistical analysis

SPSS V25 (SPSS Inc., Chicago, Illinois) was used for statistical testing. Summary data are presented as mean ± standard deviation or median [25–75% inter‐quartile range (IQR) or range]. Between group differences were compared using the *t*‐test, Mann–Whitney *U*‐test, and the *χ* ^2^ test, as appropriate. BNP, creatinine, eGFR, and all plasma biomarkers were log_10_ transformed before analysis. 6MWT distance was square root transformed.

Spearman's correlations were performed to check for potential associations of FGF23 with continuous variables in HFpEF patients. Multivariable linear regression modelling was undertaken to determine variables independently associated with 6MWT distance. *P* < 0.05 was considered significant.

Event rates were calculated from Kaplan–Meier analysis. Differences in survival curves were tested using the log‐rank test. To account for missing data (iECV in a small minority as reported previously^12^), imputation was undertaken five times, and results were averaged in agreement with Rubin's rules during survival analysis. Cox proportional hazards analysis was initially undertaken to identify baseline variables associated with the composite endpoint. Individual covariates associated with the endpoint at *P* < 0.1 were then entered into multivariable analysis to identify independent predictors using both backwards and forwards stepwise elimination methods. Four separate, clinically relevant multivariable models were generated including a final model incorporating the strongest predictors. Generally, multivariable models were limited to no more than six parameters, allowing for approximately one parameter/10 composite events. The final model however was also adjusted for additional, well‐recognized prognosticators [e.g. blood pressure (BP) and NYHA class] and potential confounders (renal function and inflammatory biomarkers), independent of their *P* values. Continuous variables were *Z*‐standardized to enable comparison of hazard ratios (HRs) based upon one standard deviation increase in the predictor variable. Receiver operator characteristic (ROC) analysis was undertaken to gauge the accuracy of the final independent Cox model to predict adverse events.

## Results

**Figure 1:**
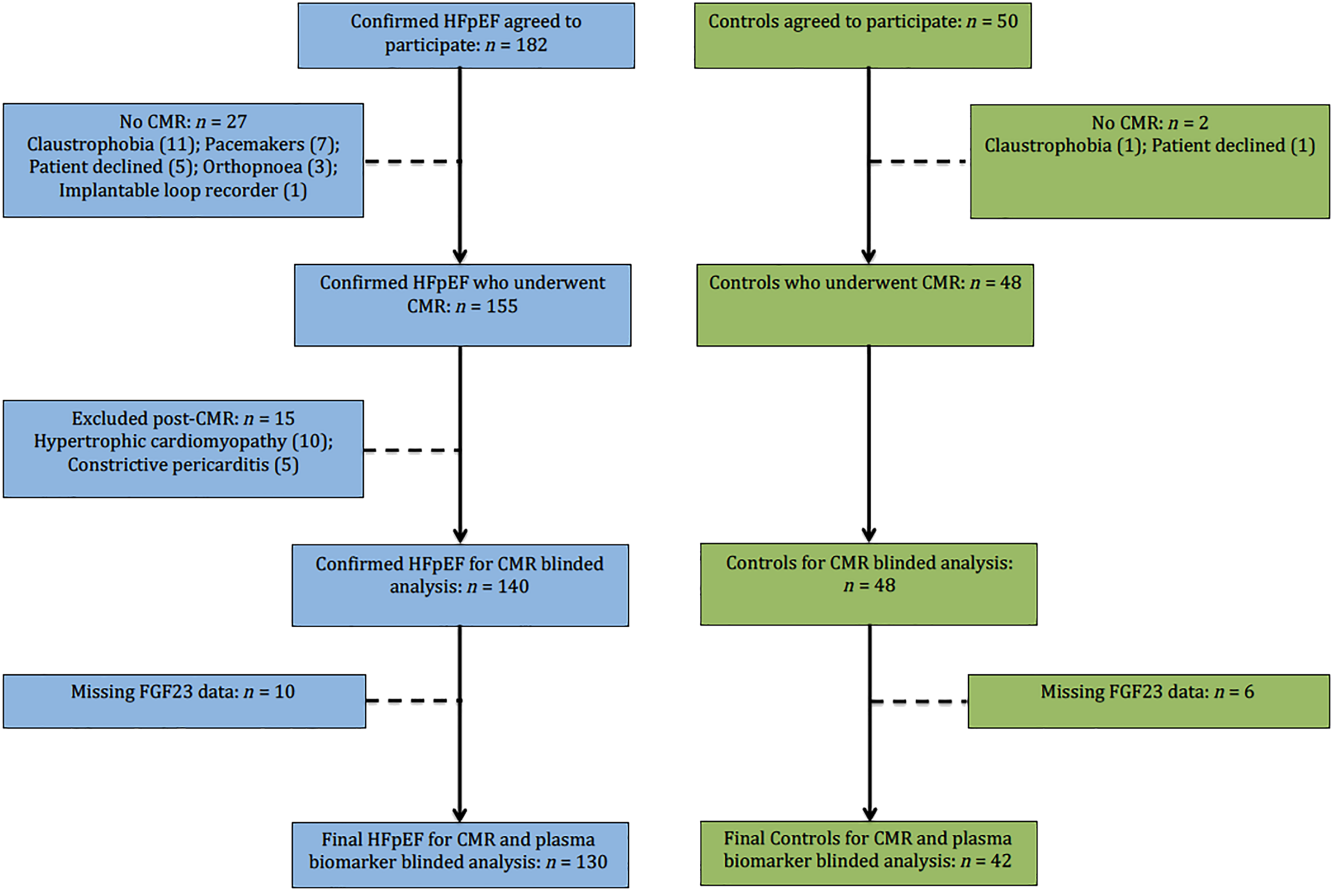
Study overview. Consort diagram illustrating recruitment, reasons for exclusion, and missing data. CMR, cardiovascular magnetic resonance imaging; HFpEF, heart failure with preserved ejection fraction.

The overall study recruitment is shown in *Figure* *1*. Following CMR, 15 patients with HFpEF were newly diagnosed with HCM or constrictive pericarditis and excluded from further analysis.^13^ Of the remaining 188 study subjects who underwent CMR, 16 had missing FGF23 data (HFpEF *n* = 10, controls *n* = 6). The final cohort with complete plasma biomarker profiles comprised 130 HFpEF patients and 42 controls. As reported previously, ECV and iECV could not be calculated in a small subset of consecutive HFpEF (*n* = 42, 32%) and controls (*n* = 3, 7%) owing to unavailability of the T1 mapping sequence at the time of the CMR scan.^12^

### Heart failure with preserved ejection fraction compared with controls

Baseline clinical and imaging characteristics are shown in *Tables* *1* and *2* respectively. Both HFpEF and controls exhibited similar age (73 years) and gender split (male 50%). HFpEF was characterized by a high prevalence of obesity, hypertension, diabetes, and atrial fibrillation. NYHA class III/IV symptoms (31%), angina (17%), and lung disease (15%) were noted in significant minorities of HFpEF.

**Table 1:** Baseline clinical characteristics

	Overall HFpEF *n* = 130	Controls *n* = 42	*P* value	HFpEF ≤ median FGF23 (≤62 pg/mL) *n* = 65	HFpEF > median FGF23 (>62 pg/mL) *n* = 65	*P* value
Age (years)	73 ± 10	73 ± 5	0.467	71 ± 9	74 ± 10	0.094
Male (%)	65 (50)	21 (50)	0.871	31 (48)	34 (52)	0.599
Heart rate (b.p.m.)	70 ± 14	68 ± 11	0.328	68 ± 13	73 ± 14	0.055
Systolic BP (mmHg)	145 ± 25	150 ± 23	0.290	151 ± 24	140 ± 25	0.010
Diastolic BP (mmHg)	74 ± 12	79 ± 10	0.015	77 ± 13	73 ± 12	0.064
Body mass index (kg/m^2^)	34 ± 7	25 ± 3	<0.0001	34 ± 7	34 ± 7	0.596
Prior HF hospitalization (%)	85 (65)	NA	NA	37 (57)	48 (74)	0.043
Atrial fibrillation (%)	41 (32)	0 (0)	<0.0001	12 (18)	29 (45)	0.001
Diabetes (%)	65 (50)	0 (0)	<0.0001	29 (45)	36 (55)	0.219
Hypertension (%)	118 (91)	21 (50)	<0.0001	57 (88)	61 (94)	0.226
Angina (%)	22 (17)	0 (0)	0.005	8 (12)	14 (22)	0.160
Known MI (%)	13 (10)	0 (0)	<0.0001			
Asthma or COPD (%)	20 (15)	3 (7)	0.111	8 (12)	12 (19)	0.331
Smoking (%)	68 (52)	16 (38)	0.079	31 (48)	37 (57)	0.292
Hypercholesterolaemia (%)	63 (49)	18 (43)	0.466	30 (46)	33 (51)	0.599
TIA or CVA (%)	15 (12)	0 (0)	<0.0001	8 (12)	7 (11)	0.859
Beta‐blocker (%)	89 (69)	2 (5)	<0.0001	44 (68)	45 (69)	0.850
ACEi or ARB (%)	112 (86)	9 (21)	<0.0001	56 (86)	56 (86)	1.000
Aldosterone antagonist (%)	41 (32)	0 (0)	<0.0001	21 (32)	20 (31)	0.850
Loop diuretic (%)	105 (81)	0 (0)	<0.0001	47 (72)	58 (89)	0.014
NYHA III/IV (%)	40 (31)	NA	NA	15 (23)	25 (38)	0.043
6 min walking test distance (m)	180 (120–260)	380 (350–440)	<0.0001	220 (159–300)	160 (105–220)	0.014
MLHF score	49 (25–64)	NA	NA	43 (20–66)	52 (33–63)	0.080
Sodium (mmol/L)	139 ± 3	140 ± 2	0.066	139 ± 4	139 ± 3	0.533
Urea (mmol/L)	9 ± 4	6 ± 2	0.0001	8 ± 3	9 ± 4	0.037
Creatinine (mmol/L)	89 (73–114)	69 (56–86)	<0.0001	79 (69–94)	100 (84–119)	0.001
eGFR	68 (53–83)	90 (77–100)	<0.0001	77 (56–90)	57 (46–75)	<0.0001
Haemoglobin (g/L)	129 ± 22	140 ± 15	<0.0001	132 ± 22	126 ± 23	0.127
BNP (pg/mL)	140 (66–258)	33 (24–43)	<0.0001	134 (51–252)	145 (80–270)	0.419
OPG (pg/mL)	100 (77–125)	92 (68–105)	0.033	89 (72–111)	109 (88–137)	<0.0001
hs‐CRP (ng/mL)	43 169 (14 992–78 805)	6914 (3531—17 393)	<0.0001	28 662 (12 620–64 390)	60 772 (20 581–95 156)	0.008
MPO (ng/mL)	212 (160–262)	153 (130–178)	<0.0001	178 (130–235)	240 (177–271)	<0.0001
FGF23 (pg/mL)	62 (42–105)	34 (22–41)	<0.0001	42 (34–51)	105 (80–177)	<0.0001

ACEi, angiotensin‐converting enzyme inhibitor; ARB, angiotensin II receptor blocker; BNP, B‐type natriuretic peptide; BP, blood pressure; b.p.m., beats per minute; COPD, chronic obstructive pulmonary disease; CVA, cerebrovascular accident; FGF23, fibroblast growth factor 23; HFpEF, heart failure with preserved ejection fraction; hs‐CRP, highly sensitive C‐reactive protein; MI, myocardial infarction; MLHF, Minnesota Living with Heart Failure questionnaire; MPO, myeloperoxidase; NYHA, New York Heart Association class; OPG, osteoprotegerin; TIA, transient ischaemic attack.

Values are mean ± SD, *n* (%) or median, inter‐quartile range. The *P* values are for the *t*‐test or *χ* ^2^ test.

**Table 2:** Baseline imaging characteristics

	Overall HFpEF *n* = 130	Controls *n* = 42	*P* value	HFpEF ≤ median FGF23 (≤62 pg/mL) *n* = 65	HFpEF > median FGF23 (≤62 pg/mL) *n* = 65	*P* value
Echocardiography						
E/e'	13 ± 5	9 ± 3	<0.0001	12 ± 5	14 ± 5	0.016
CMR						
LV						
LVEF (%)	56 ± 5	58 ± 5	0.048	57 ± 5	55 ± 5	0.294
LVEDV (mL)	160 ± 41	145 ± 33	0.033	166 ± 40	156 ± 44	0.163
LVEDVI (mL/m^2^)	79 ± 18	80 ± 14	0.565	82 ± 18	76 ± 19	0.054
LVESV (mL)	71 ± 23	62 ± 18	0.016	73 ± 23	70 ± 24	0.455
LVESVI (mL/m^2^)	35 ± 10	34 ± 8	0.511	36 ± 11	34 ± 10	0.265
LVM (g)	108 ± 33	81 ± 21	0.0001	109 ± 37	108 ± 31	0.842
LVMI (g/m^2^)	52 ± 15	45 ± 9	<0.0001	53 ± 16	52 ± 14	0.465
LV mass/LV volume	0.68 ± 0.16	0.57 ± 0.09	<0.0001	0.66 ± 0.15	0.69 ± 0.17	0.228
LA						
LAVmax (mL)	106 ± 47	61 ± 22	<0.0001	104 ± 47	111 ± 48	0.357
LAVImax (mL/m^2^)	53 ± 25	34 ± 12	<0.0001	52 ± 24	55 ± 27	0.410
LAVmin (mL)	77 ± 48	30 ± 13	<0.0001	71 ± 48	85 ± 50	0.101
LAVImin (mL/m^2^)	38 ± 26	17 ± 7	<0.0001	35 ± 24	42 ± 27	0.126
LAEF (%)	32 ± 16	51 ± 10	<0.0001	36 ± 16	28 ± 15	0.004
Tissue characterization						
ECV (%)	28 ± 5	25 ± 3	<0.0001	27 ± 4	28 ± 5	0.418
iECV	14 ± 4	11 ± 2	<0.0001	13 ± 4	14 ± 5	0.329
LGE positive – MI (%)	20 (15)	0 (0)	0.004	7 (11)	13 (20)	0.145
LGE positive – non‐MI (%)	47 (36)	5 (12)	0.001	24 (37)	23 (35)	0.855

Abbreviations as per *Table* *1*.

ECV, extracellular volume; EDVI, end‐diastolic volume indexed; EF, ejection fraction; ESVI, end‐systolic volume indexed; iECV, indexed ECV; LAVImax, maximal left atrial volume indexed; LGE, late gadolinium enhancement; LV, left ventricle; LVMI, left ventricular mass indexed.

Compared with controls, HFpEF was characterized by markedly lower exercise capacity [6MWT distance (median 180 m vs. 380 m; *P* < 0.0001)], lower haemoglobin, worse renal function, increased LV mass, and more concentric LV remodelling (mass/volume). Surrogate markers of diastolic dysfunction, that is, echocardiographic E/e′, BNP, maximal left atrial volume index [LAVImax], and LAEF were worse in HFpEF. Focal (MI and non‐MI) and diffuse (ECV and iECV) fibrosis were greater in HFpEF. Plasma biomarkers associated with inflammation (hs‐CRP, MPO) and OPG were also higher in HFpEF (*Table* 1).

### Plasma fibroblast growth factor 23 in heart failure with preserved ejection fraction

FGF23 levels were significantly higher in HFpEF compared with controls [62 (42–105) vs. 34 (22–41) pg/mL, *P* < 0.0001] and irrespective of whether controls were hypertensive or normotensive (*P* < 0.0001 for each). FGF23 levels did not differ significantly between hypertensive and non‐hypertensive controls (*P* = 0.920).

HFpEF baseline characteristics stratified according to median FGF23 (62 pg/mL) are also shown in *Tables* 1 and 2. HFpEF patients with FGF23 above the median plasma concentration experienced lower systolic BP, more frequent prior HF hospitalization, more prevalent AF, greater prescription of loop diuretics, worse renal function (urea, creatinine, and eGFR), worse exercise capacity (greater proportion of NYHA III/IV and reduced 6MWT distance), higher LV filling pressures (E/e′), and lower LAEF. MLHF scores were worse in the higher FGF23 group, albeit statistical significance was not reached (*P* = 0.080). HFpEF patients with above‐median FGF23 showed higher levels of plasma hs‐CRP, MPO, and OPG. There were no significant differences in CMR measured LV or LA volumes and function or LV mass and fibrosis according to median FGF23 in HFpEF.

The results of FGF23 correlation analyses in HFpEF are shown in *Table* *3*. Moderate strength correlations were noted with clinical parameters. Higher FGF23 correlated with the following: lower systolic and diastolic BP, more advanced NYHA class, lower haemoglobin and E/e′, lower 6MWT distance, poorer renal function, and higher plasma levels of hs‐CRP, MPO, and OPG. No significant associations were noted between FGF23 and any CMR parameter with the exception of a weak correlation (*β* − 0.194, *P* = 0.030) with LV end‐diastolic volume indexed to BSA (LVEDVI). Specifically, FGF23 did not correlate with CMR measures of established association with prognosis: ECV (*r* = 0.116, *P* = 0.280), iECV (*r* = 0.031, *P* = 0.778), LAVImax (*r* = 0.023, *P* = 0.795), LAVImin (*r* = 0.093, *P* = 0.293), and LAEF (*r* = 0.166, *P* = 0.058).

**Table 3:** Significant associations of FGF23 with other continuous variables

	Correlation coefficient (Spearman's)	*P* value
Clinical		
Systolic BP	−0.255	0.004
Diastolic BP	−0.211	0.017
NYHA	0.308	<0.0001
Haemoglobin	−0.265	0.002
Square root transformed 6MWT distance	−0.345	<0.0001
Blood		
Urea	0.267	0.002
Lg creatinine	0.351	<0.0001
Lg eGFR	−0.367	<0.0001
OPG	0.446	<0.0001
hs‐CRP	0.207	0.018
MPO	0.311	<0.0001
Imaging		
E/e'	0.298	0.001
LVEDVI	−0.194	0.030

**Figure 2:**
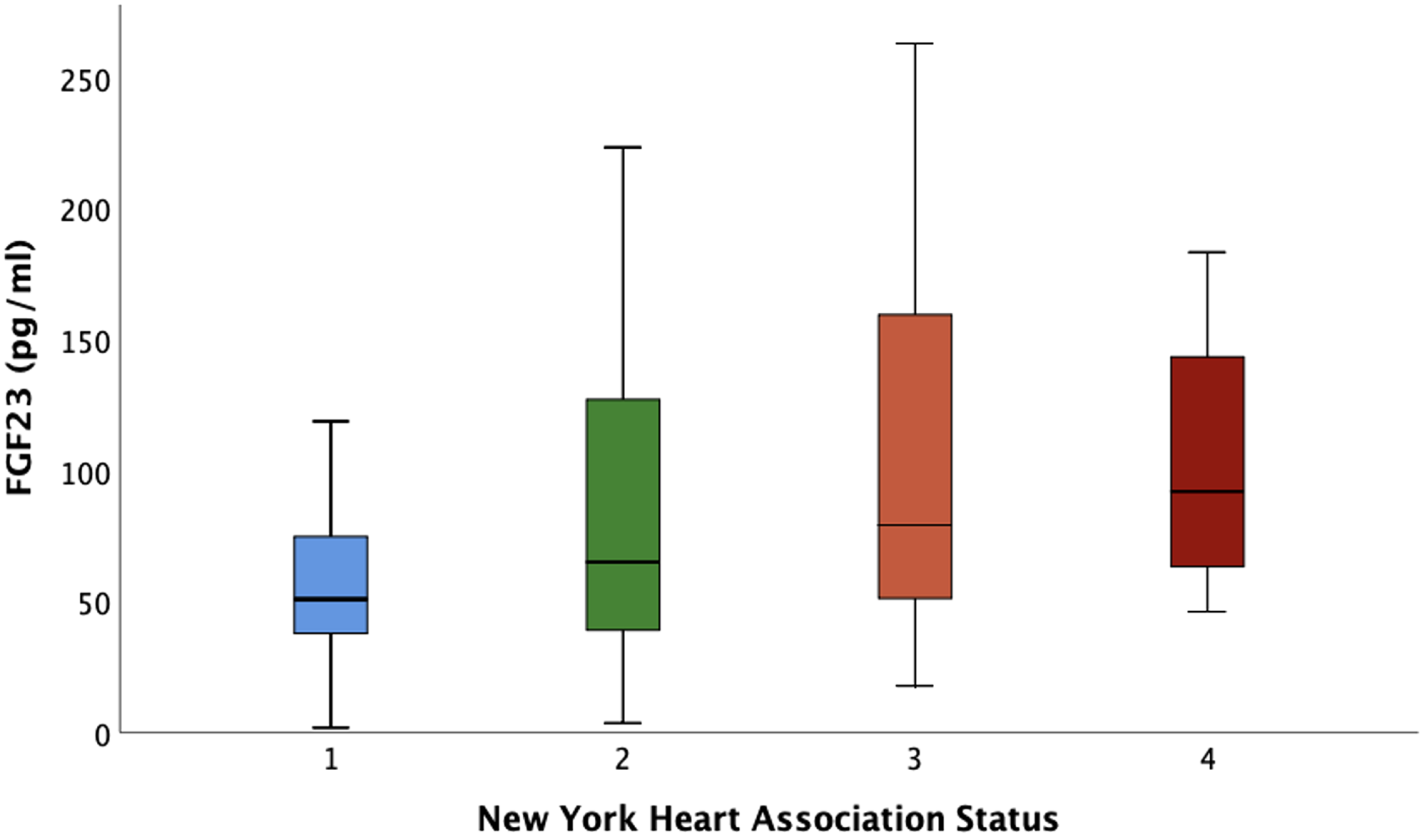
Association of fibroblast growth factor 23 with New York Heart Association status.

FGF23 levels increased with worsening NYHA status (*Figure* *2*). The results of univariable and multivariable linear regression modelling for 6MWT distance are shown in *Table* *4*. Creatinine, eGFR, LVEDVI, and indexed LV end‐systolic volume (LVESVI) exhibited collinearity. Overall, FGF23 (*β* − 0.198, *P* = 0.012), age (*β* − 0.343, *P* = 0.0001), body mass index (BMI) (*β* − 0.397, *P* = 0.0001), and haemoglobin (*β* 0.213, *P* = 0.006) were independently associated with 6MWT distance. The final multivariable model yielded the following values; *R* = 0.592, *R* ^2^ = 0.351, adjusted *R* ^2^ = 0.328. The *R* ^2^ and adjusted *R* ^2^ values for the contribution of FGF23 towards the final model were 0.121 and 0.114, respectively. The relationship between FGF23 and 6MWT distance is illustrated in *Figure* *S1*.

**Table 4:** Univariable and multivariable linear regression models for the associations with 6 min walking test

	Univariable analysis		Multivariable analysis	
	Standardized coefficients (*β*)	*P* value	Standardized coefficients (*β*)	*P* value
Clinical				
Age	−0.294	0.001	−0.343	0.0001
Gender (male)	0.197	0.029		
Diastolic BP	0.225	0.012		
BMI	−0.323	0.0001	−0.397	0.0001
Blood				
Lg creatinine^a^	−0.189	0.037		
Lg eGFR^a^	0.298	0.001		
Haemoglobin	0.253	0.005	0.213	0.006
Lg BNP	−0.181	0.045		
Lg FGF23	−0.342	0.0001	−0.198	0.012
Imaging				
E/e'	−0.222	0.019		
LVEDVI^a^	0.221	0.016		
LVESVI^a^	0.154	0.097		

Model *R* = 0.592, *R* ^2^ = 0.351, adjusted *R* ^2^ = 0.328.

^a^ Variables that exhibited significant co‐linearity; of these, Lg eGFR and LVEDVI were entered into multivariable analysis.

### Survival analysis

During median follow‐up of 1428 days (IQR 1153–1663), there were 61 first clinical events (21 deaths and 40 HF hospitalizations) in patients with HFpEF. No events were observed in the control group.

### Cox regression analysis

Sixteen variables showed univariate association with adverse outcomes (*Table* *5*). Clinical variables were as follows: greater age, prior HF hospitalization, NYHA III/IV symptoms, lower diastolic BP, and 6MWT distance. Laboratory indices associated with increased risk of end points were as follows: lower haemoglobin and eGFR, and higher levels of creatinine, BNP, and FGF23. Imaging parameters included higher E/e′, LV mass indexed to BSA (LVMI), LAVImax, ECV and iECV, and lower LAEF. Of these, eGFR and ECV were excluded from multivariable analysis due to co‐linearity. On multivariable analysis (*Table* *S1*), FGF23 remained associated with outcome in three separate models incorporating clinical factors, blood sampling, and imaging parameters. In a final model comprising the strongest predictors overall as well as diastolic BP, NYHA class, Lg creatinine, Lg hs‐CRP, and Lg MPO, FGF23 [adjusted HR 1.665; 95% confidence interval (CI) 1.284–2.160; *P* < 0.0001] remained an independent predictor along with Lg BNP (HR 1.433, CI 1.053–1.951; *P* = 0.022) and prior HF hospitalization (HR 2.058; CI 1.074–3.942; *P* = 0.030). Plots of the scaled Schoenfeld residuals against time were centred around zero with no time‐dependent trend, suggesting that the proportional hazards assumption was upheld using the functional forms supplied to the Cox models. The final independent Cox model to predict outcomes yielded and area under the ROC curve of 0.786 (*P* < 0.0001).

**Table 5:** Univariable predictors for the composite endpoint of death and/or hospitalization with heart failure

	Univariable predictors of outcome	
	Hazard ratio (95% CI)	*P* value
Clinical		
Age	1.346 (1.047–1.730)	0.020
Prior HF hospitalization	2.745 (1.456–5.175)	0.002
Diastolic BP	0.645 (0.483–0.861)	0.003
NYHA 3/4	1.649 (0.983–2.763)	0.058
Square root transformed 6MWT	0.720 (0.536–0.969)	0.030
Clinical blood samples		
Haemoglobin	0.733 (0.570–0.941)	0.015
Lg creatinine	1.328 (1.045–1.689)	0.020
Lg eGFR^a^	0.821 (0.660–1.020)	0.075
Lg BNP	1.565 (1.132–2.164)	0.007
Lg FGF23	1.732 (1.356–2.211)	<0.0001
Imaging		
E/e′	1.504 (1.180–1.918)	0.001
LVMI	1.305 (1.002–1.699)	0.048
LAVImax	1.266 (1.012–1.582)	0.039
LAEF	0.762 (0.590–0.984)	0.037
ECV^a^	1.612 (1.136–2.287)	0.008
iECV	1.732 (1.151–2.606)	0.008

^a^ Parameters not entered into Cox regression multivariable modelling due to co‐linearity.

### Kaplan–Meier analysis

Kaplan–Meier survival curves stratified according to median FGF23 are shown in *Figure* S2. Above‐median FGF23 levels in HFpEF were associated with markedly elevated risk of adverse outcome (HR 3.171, CI 1.544–6.510, log‐rank *P* < 0.0001).

## Discussion

In this extensively phenotyped cohort of patients with HFpEF, higher FGF23 was associated with more severe HF, as evidenced by higher NYHA class and BNP, lower systolic and diastolic BP, and higher LV filling pressure (E/e′). Furthermore, higher FGF23 was associated with evidence of more advanced anaemia and renal dysfunction, as well as with evidence of systemic inflammation. Importantly, we observed independent associations between higher circulating FGF23 concentrations and exercise capacity (6MWT), and the risk of death or hospitalization with HF in patients with HFpEF.

FGF23 is a regulator of bone mineral, vitamin D, and iron homeostasis; in turn, FGF23 production is regulated by various factors including iron deficiency and inflammation.^18^ Elevated FGF23 levels are seen in chronic kidney disease, where they are associated with adverse outcome^5^ and in cohorts with stable ischaemic heart disease^19^ and HFrEF.^20^ There are very few prior reports relating to FGF23 in HFpEF. In the TIME CHF study, FGF23 was found to be higher in HFpEF than in HFrEF but was not associated with risk of death or HF hospitalization.^21^ Similarly, FGF23 was not associated with mortality risk in patients undergoing coronary angiography in the Ludwigshafen Risk and Cardiovascular (LURIC) Health Study.^22^ While our observations of increased risk of adverse outcome in association with elevated FGF23 appear to contrast with those of TIME CHF and LURIC,^21^, ^22^ the former included only 73 patients with HFpEF, and neither was a specific study of HFpEF. Our observed association between circulating FGF23 and risk of death or HF hospitalization in HFpEF confirms similar findings from a retrospective analysis of the Treatment of Preserved Cardiac Function Heart Failure with an Aldosterone Antagonist (TOPCAT) Trial^23^ as well as from a recently published prospective, observational study.^24^ Furthermore, our outcome data benefit from substantially longer follow‐up: median 44 vs. 34 and 30 months.

The rationale for the link between FGF23 and adverse outcomes in HFpEF is not clear. It is known that FGF23 is associated with prognosis in, and progression of, chronic jkidney disease^5^ and with risk of incident HF in community‐based populations^6^ and in patients with HFrEF.^7^ A direct causative link between FGF23 and adverse outcomes is supported by the established link between FGF23 and LV remodelling in clinical studies^25^, ^26^ and the induction of LVH by FGF23 in animal models,^8^, ^9^ an effect attenuated by blockade of the FGFR4 receptor.^10^ While it may be postulated that FGF23 is associated with prognostic risk via its association with renal impairment,^5^ our data indicate that FGF provides information over and above knowledge of renal function, as indicated by the independent association of both FGF23 and parameters of renal function in multivariate association with outcomes.

Our observation of association between circulating FGF23 and adverse outcome in HFpEF is in keeping with that from a recent study^24^ of similar sample size as our cohort. Similar to our study, these authors found FGF23 to be higher in HFpEF compared with controls and to be associated with plasma NT‐proBNP levels and more prevalent atrial fibrillation. Moreover, as in the present report, the previous study found FGF23 levels to be associated with risk of all‐cause death or HF hospitalization. While our study did not find association with ECV in contrast to the previous report,^24^ we suggest there are consistent observations in the two studies, with differences in the characteristics of the populations, in imaging techniques, and in parameters considered in multivariate analyses, all likely to contribute to variation in the strength of observed associations.

Importantly, to the association with adverse prognosis, we report an additional association for FGF23 with reduced exercise capacity in HFpEF. HFpEF is a syndrome characterized by limitation of exercise capacity, in the presence of preserved LVEF, LVH, and elevated plasma natriuretic peptides. Our cohort of 130 patients demonstrated the archetypal HFpEF phenotype, with LVEF comparable with the matched control cohort (56 vs. 58%), moderate elevation of BNP, and clearly elevated LV mass and filling pressures. Exercise capacity in patients with HFpEF was reduced markedly, with median 6MWT distance (180 m) less than half of that compared with age‐matched and sex‐matched controls (vs. 380 m).

A number of clinical variables demonstrated univariate association with 6MWT distance, including parameters of renal impairment (eGFR and creatinine) and plasma FGF23 (*Table* 3). In this context, the independent association with lower 6MWT distance of greater age**,** lower haemoglobin, higher BMI, and higher FGF23, but not of eGFR or creatinine, is of note. As with the association with prognosis, we cannot ascribe a causal relationship between elevated FGF23 and reduced 6MWT distance. However, potential contributors to this apparent effect include the potential for FGF23 to induce LVH, and its association with progressive renal impairment, as already noted. However, using gold standard CMR imaging, we failed to observe any correlation between indices of FGF23 with LV mass indices. Alternatively, FGF23 may promote myocardial fibrosis and remodelling via activation of the renin–angiotensin–aldosterone system^27^ and may contribute to the development of anaemia^28^; we did observe a relatively strong inverse association between FGF23 and haemoglobin and treatment of iron deficiency in HF is known to result in reduction in FGF23 levels.^29^ Whether blockade of FGF23 would result in improvement in parameters of haemoglobin and indeed of LVH in HFpEF is not known.

With regard to exercise capacity in HFpEF, we note the lack of multivariate association with 6MWT distance for any imaging parameter. While elevated filling pressure (E/e′) and measures of LV remodelling (LVEDVI and LVESVI) showed univariate associations, these were not retained after adjustment for covariates. As well as higher age and BMI and lower haemoglobin, higher FGF23 was the only laboratory parameter to retain a multivariate association with poorer exercise capacity. We suggest that this observation supports a possible direct link between FGF23 and exercise capacity in this patient group.

FGF23 plays a pivotal role in bone mineral homeostasis, and levels increase in response to increasing phosphate levels and increase as renal impairment progresses. In this context, we noted elevated circulating levels of a second biomarker related to mineral metabolism and OPG in our patients with HFpEF, with a relatively strong correlation between FGF23 and OPG levels (*r* = 0.446, *P* < 0.0001). Clustering of these peptides as markers of outcome in HFpEF has been reported in one previous study,^23^ the relevance of elevated markers of bone mineral metabolism in HFpEF merits further investigation.

### Potential implications of our study

Our data indicate that FGF23 appears to be associated with a more severe clinical phenotype of HFpEF, conferring additional prognostic information beyond standard clinical characteristics and enables patients to be stratified into high‐risk and low‐risk groups. Furthermore, FGF23 appears intimately involved in pathways of inflammation, serving as a potential therapeutic target and also as a biomarker of treatment response.

### Limitations

Our study has a number of strengths and weaknesses. Our patients with HFpEF were phenotyped in detail with CMR imaging complementing transthoracic echo and detailed biomarker assessment. Importantly, our patients underwent standardized 6MWT assessment of exercise capacity. Together, our observations of association of FGF23, and the lack of association of CMR imaging parameters, with exercise capacity can be regarded as relatively robust. However, we acknowledge that these associations should be assessed in additional populations of patients with HFpEF. The overall study sample size is relatively small, and the limited number of events is also a limitation. While FGF23 levels were only measured at baseline in our cohort, repeat plasma sampling over time may offer improved precision for the estimation of risk. The single‐centre nature of our study is a clear potential weakness. Furthermore, we do not have serum phosphate or iron levels. While we observed associations between FGF23 and a number of clinical parameters, and with well‐defined end points, we are not able to ascribe a cause‐and‐effect relationship with risk of death or HF hospitalization. However, we have outlined possible pathways by which FGF23 may be directly related to these events, and indeed to reduce exercise capacity in HFpEF, which merit further investigation in future clinical studies in the increasingly prevalent HFpEF population.

## Conclusions

In conclusion, FGF23 levels are elevated in patients with HFpEF compared with age‐matched and sex‐matched control subjects and are associated with reduced exercise capacity and increased risk of death or HF. Future studies of possible mechanistic links between FGF23 and prognosis in HFpEF are merited.

## Conflict of interest

None declared.

## Funding

This work was supported by the National Institute for Health Research (NIHR Leicester Cardiovascular Biomedical Research Centre, overall project grant IRS_BRU_0211_20033) and the John and Lucille Van Geest Foundation. GPM was supported by a National Institute for Health Research Career Development Fellowship (2014‐07‐045) and Research Professorship (RP‐2017‐08‐ST2‐007).

## Author contributions

PK recruited the patients, supervised the study visits and CMR scans (with AS and JNK), analysed the data including qualitative analysis for the presence of MI, performed the statistical analysis, and drafted the initial manuscript along with IBS and JRA. GGS undertook follow‐up outcome data collection. BNP and other serum sampling were undertaken in the hospital pathology laboratory under the supervision of PG. PK, IBS, LLN, and GPM conceived the study. All authors critically revised the manuscript for important intellectual content, approved the final version for submission, and agreed to be accountable for all aspects of the work in ensuring that questions relating to the accuracy or integrity of any part of the work are appropriately investigated and resolved.

## Ethics statement

The study complied with the Declaration of Helsinki and the National Research Ethics Service approved the study. Written informed consent was obtained from all subjects prior to participation.

## Supporting information


**Figure S1.** Association of fibroblast growth factor 23 with six minute walk test distance.
Caption: Scatter plot illustrating the relationship between Log fibroblast growth factor 23 with square root transformed six minute walk test distance.

**Figure S2.** Kaplan–Meier survival analysis.
Caption: Survival curves stratified according to median fibroblast growth factor 23.

**Table S1.** Multivariable predictor models inclusive of FGF23 for the composite endpoint of death and/or hospitalization with heart failure.
