# Supplementary figures and images for: Fibroblast‐growth‐factor‐23 in heart failure with preserved ejection fraction: relation to exercise capacity and outcomes
Source: ESC Heart Fail. 2020 Sep 16;7(6):4089–99. doi: 10.1002/ehf2.13020 (PMC7755022; doi:10.1002/ehf2.13020)

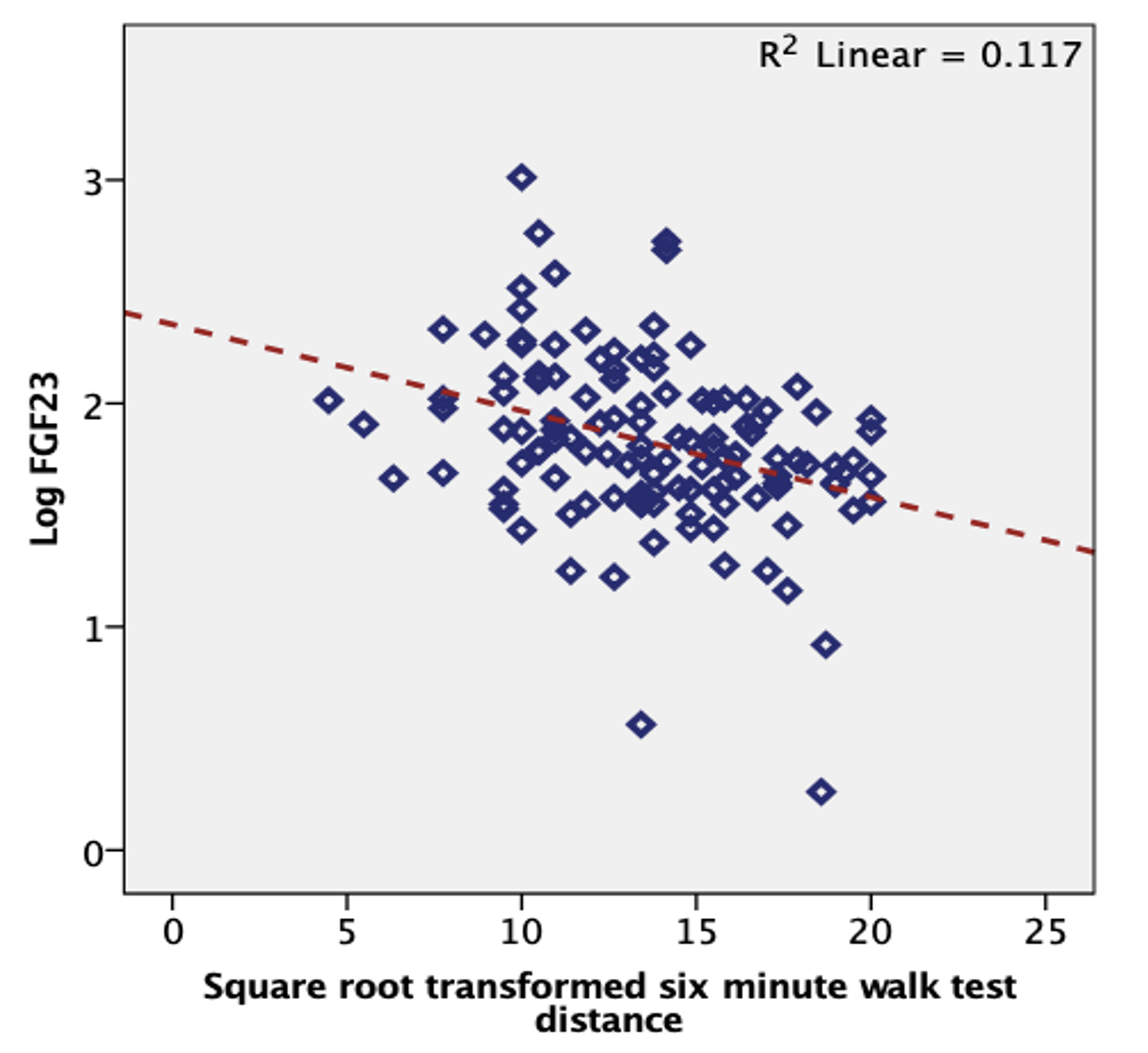

Supplement: Supplementary file 1 — Figure S1. Association of fibroblast growth factor 23 with six minute walk test distance. Caption: Scatter plot illustrating the relationship between Log fibroblast growth factor 23 with square root transformed six minute walk test distance. [file EHF2-7-4089-s001.tiff]

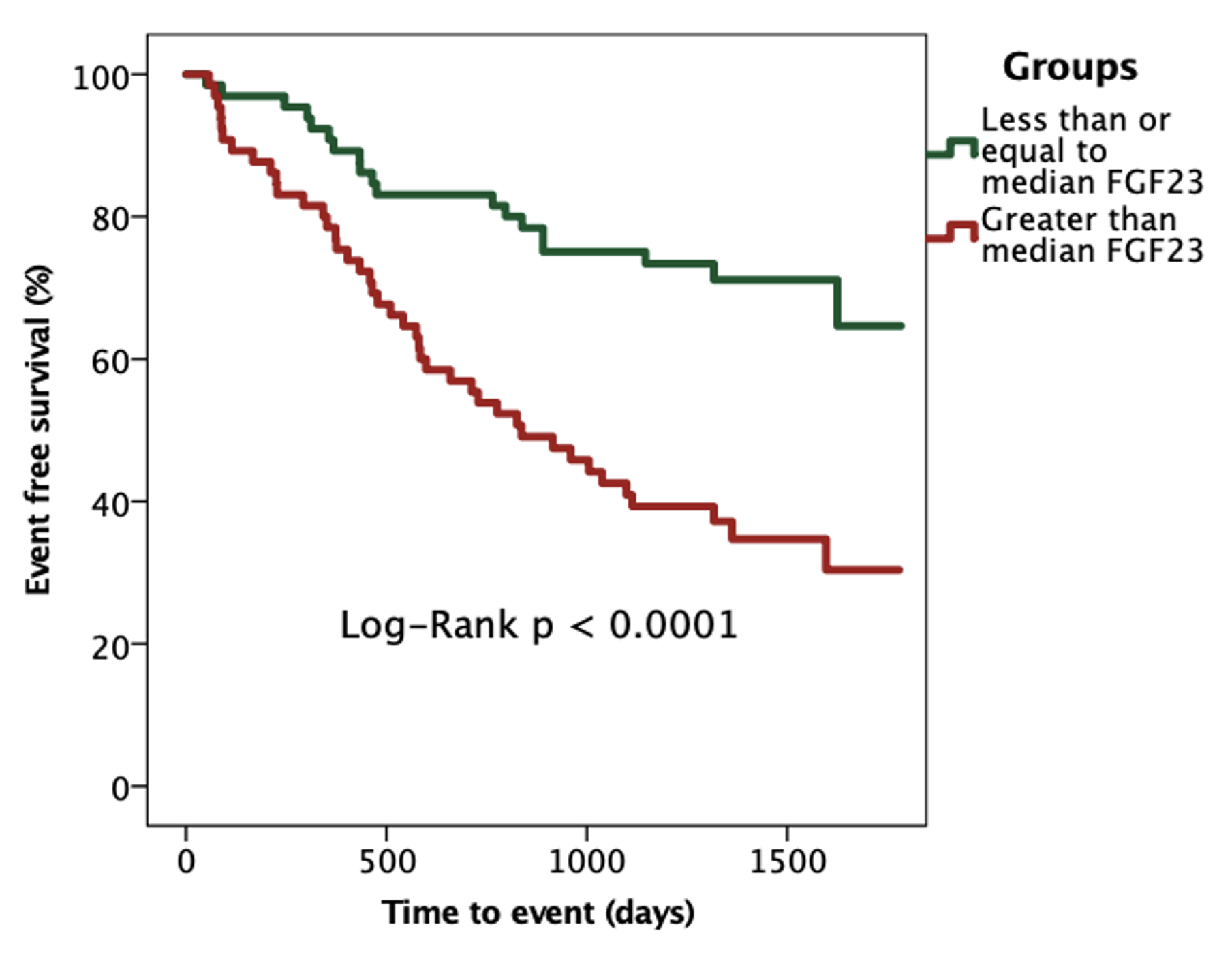

Supplement: Supplementary file 2 — Figure S2. Kaplan–Meier survival analysis. Caption: Survival curves stratified according to median fibroblast growth factor 23. [file EHF2-7-4089-s002.tiff]
