# Supplementary material for: Fibroblast‐growth‐factor‐23 in heart failure with preserved ejection fraction: relation to exercise capacity and outcomes
Source: ESC Heart Fail. 2020 Sep 16;7(6):4089–99. doi: 10.1002/ehf2.13020 (PMC7755022; doi:10.1002/ehf2.13020)
Supplement: Supplementary file 3 — Table S1. Multivariable predictor models inclusive of FGF23 for the composite endpoint of death and/or hospitalization with heart failure. [file EHF2-7-4089-s003.docx]

Online supplementary Table 1.

Multivariable predictor models inclusive of FGF23 for the composite endpoint of death and/or hospitalization with heart failure

| Multivariable predictors of outcome | | |
| --- | --- | --- |
|  | Hazard ratio (95% CI) | P value |
| Clinical | | |
| Age | 1.302 (1.020-1.661) | 0.034 |
| Prior HF hospitalization | 2.391 (1.259-4.540) | 0.008 |
| Diastolic BP | 0.845 (0.623-1.145) | 0.277 |
| NYHA 3/4 | 1.103 (0.494-2.461) | 0.811 |
| Square root transformed 6MWT | 1.038 (0.729-1.478) | 0.835 |
| + Lg FGF23 | 1.637 (1.259-2.128) | <0.0001 |
| Clinical blood samples | | |
| Haemoglobin | 0.927 (0.719-1.196) | 0.561 |
| Lg Creatinine | 1.082 (0.831-1.410) | 0.558 |
| Lg BNP | 1.621 (1.146-2.293) | 0.006 |
| + Lg FGF23 | 1.752 (1.360-2.258) | <0.0001 |
| Imaging | | |
| E/e’ | 1.321 (1.020-1.710) | 0.035 |
| LVMI | 1.053 (0.692-1.601) | 0.809 |
| LAVImax | 0.868 (0.534-1.409) | 0.566 |
| LAEF | 0.664 (0.504-0.875) | 0.004 |
| iECV | 1.332 (0.895-1.984) | 0.152 |
| + Lg FGF23 | 1.653 (1.263-2.163) | <0.0001 |
| Combined strongest model | | |
| Age | 1.110 (0.791-1.529) | 0.573 |
| Diastolic BP | 1.029 (0.743-1.424) | 0.864 |
| NYHA 3/4 | 0.959 (0.501-1.836) | 0.899 |
| Prior HF hospitalization | 2.058 (1.074-3.942) | 0.030 |
| Lg BNP | 1.433 (1.053-1.951) | 0.022 |
| Lg Creatinine | 1.160 (0.867-1.552) | 0.319 |
| Lg hs-CRP | 1.181 (0.880-1.585) | 0.269 |
| Lg MPO | 0.954 (0.722-1.261) | 0.743 |
| E/e’ | 1.156 (0.888-1.504) | 0.281 |
| LAEF | 0.882 (0.684-1.136) | 0.331 |
| + Lg FGF23 | 1.665 (1.284-2.160) | <0.0001 |
